# Assessment of Digital Intraoral Periapical Radiograph for the Detection of Apical Root Resorption in Inflammatory Periapical Pathologies: A Radiovisiography Study

**DOI:** 10.7759/cureus.44885

**Published:** 2023-09-08

**Authors:** Adeeba R Siddique, Mukta B Motwani, Nandkishor J Bankar

**Affiliations:** 1 Oral Medicine and Radiology, Datta Meghe Medical College, Datta Meghe Institute of Higher Education and Research, Nagpur, IND; 2 Oral Medicine and Radiology, Vidya Shikshan Prasarak Mandals Dental College and Research Center, Nagpur, IND; 3 Microbiology, Jawaharlal Nehru Medical College, Datta Meghe Institute of Higher Education and Research, Wardha, IND

**Keywords:** abscesses, granulomas, cysts, inflammatory periapical pathologies, periapical radiograph, periapical diseases, apical root resorption, radiovisiography

## Abstract

Introduction

Resorption often takes the form of external inflammatory root resorption. Apical periodontitis or an apical cyst is the most typical cause of external inflammatory root resorption. Failure of endodontic treatment can occur if severe apical root resorption occurs.This is due to the difficulty of reaching these sites.Apical root resorption is usually discovered during routine radiographs and is usually in its later stages. If the lesion is advanced, extraction is the only viable solution. An accurate diagnosis of incipient root resorption is essential. This research is designed to analyze the effectiveness of digital intraoral periapical radiographs in assessing apical root resorption (ARR) related to periapical pathologies.

Material and methods

This cross-sectional radiographic observational research was conducted in a dental college and hospital in central India. Radiovisiography (RVG) images of 190 patients’ teeth with inflammatory periapical pathologies were evaluated to determine the presence or absence of resorption in the apical area of the root. After the radiographic assessment of the apical root resorption, the extraction of the affected teeth was done under all aseptic conditions. The periapical tissue was sent for histological analysis and the extracted tooth sample was examined for the presence or absence of apical root resorption.

Results

In comparison to apical periodontitis, the proportion of severe root resorption patients was significantly higher in abscess and periapical granuloma. Using Pearson's Chi-square test, the difference in patient proportions according to the kind of resorption in the three radiological diagnosis groups was statistically significant with a p-value of 0.0058.

Conclusion

It was concluded that on radiographic examination, digital intraoral periapical radiographs were found to be accurate in determining periapical apical pathologies and apical root resorption.

## Introduction

Teeth are strong as long as their roots are safe. Any problem with the root, such as root resorption, will affect the function of the tooth [1]. Deciduous teeth exfoliate due to normal physiologic root resorption, while permanent teeth can undergo internal or external resorption [2,3]. Resorption often takes the form of external inflammatory root resorption. Apical periodontitis or an apical cyst is the most typical cause of external inflammatory root resorption [4]. Caries that penetrate the pulp, traumatic invasive luxation or avulsion with reimplantation, all cause the formation of periapical cysts and tumors as well as necrosis of the root canal system. These conditions result in external inflammatory root resorption [5].

The periapical pathologic process that results from the local inflammatory process that releases cytokines, prostaglandins, and other substances frequently has an adverse effect known as apical root resorption [6]. Apical periodontitis is asymptomatic and it leads to root resorption which affects the success of endodontic treatment. Failure of endodontic treatment can occur if severe apical root resorption occurs [7]. This is due to the difficulty of reaching these sites [1]. Apical root resorption is usually discovered during routine radiographs and is usually in its later stages. If the lesion is advanced, extraction is the only viable solution. An accurate diagnosis of incipient root resorption is essential [8]. The direct replacement of an X-ray film with an electronic image receptor is known as direct digital radiography. The advantages are near instantaneous availability of images without removing the sensor from the mouth. Direct digital radiography(DRR) also helps identify canal locations, root curvatures, and abnormalities in the periapical region. It reduces radiation dose by 80% compared to conventional intraoral periapical radiography [9]. Therefore, current research is designed to analyze the effectiveness of digital intraoral periapical radiographs in assessing apical root resorption (ARR) related to periapical pathologies.

## Materials and methods

This cross-sectional radiographic observational research was conducted in a dental college and hospital in central India after the approval from the Institutional Ethical Committee at Vidya Shikshan Prasarak Mandals Dental College and Research Center, Nagpur (approval number VSPM’S/DCRC/Dean/IEC/PG/25/2018). Radiovisiography (RVG) images of 190 patients’ teeth with inflammatory periapical pathologies were evaluated to determine the presence or absence of resorption in the apical area of the root. Patients with inflammatory periapical diseases, such as periapical cysts, periapical granulomas, and periapical abscesses, have been diagnosed clinically and radiologically. Patients with teeth that cannot be saved conservatively and willing to undergo extraction of the teeth involved with periapical pathologies were included in the study. Patients suffering from systemic diseases such as hyperparathyroidism, hypoparathyroidism, hypophosphatemia, hyperphosphatemia, unwilling to extract the affected teeth with periapical lesions, decayed teeth that can be saved conservatively, patients who have undergone orthodontic treatment and with advanced periodontitis were excluded from the study. After obtaining written informed consent from the patients, they were examined clinically, and detailed case histories were recorded.

Radiographic evaluation

Using a Carestream RVG 5200 sensor (Carestream Dental LLC, Atlanta, GA) and CS imaging software version 7 (Carestream Dental LLC, Atlanta, GA), digital intraoral periapical radiographs of patients with periapical disorders were taken, while all required safety precautions were taken. The acquisition protocol used was 70 kvp, 8 mA, and 12-bit scale. Evaluations were done on the lamina dura continuity, periodontal ligamental space size, and root surface morphology.

Criteria to assess the severity of apical root resorption

Advanced imaging processing tools of Carestream RVG 5200 sensor with CS imaging software version 7 with acquisition protocol of 70 kvp, 8 mA, and 12-bit scale with all protective measures were then used to assess the teeth with periapical radiolucencies for the presence or absence of resorption in the apical part of the root, based on the following standards: No resorption, if the contour of the root surface is intact and has a consistent density. Moderate resorption is present if the apical root contour contains blurring irregularities and less radioactively rich regions. There is severe resorption if there are obvious radiolucent indentations or shortening of the root tip.

After radiographic assessment of the apical root resorption by the three observers, extraction of affected teeth was done under all aseptic conditions. The periapical tissue was removed by scraping and then collecting it in a wide-mouthed bottle with 10% neutral buffered formalin. The extracted tooth sample was evaluated for the presence or absence of apical root resorption and the periapical tissue was sent for histopathological examination [10].

Statistical analysis

The statistical significance of the difference in apical root resorption (ARR) prevalence between pathologies was determined using Pearson’s Chi-square test. The analysis was performed using SPSS ver 20.0 (IBM Corp., Armonk, NY).

## Results

Table 1 reveals how the patients are distributed in terms of age. Maximum, or 101 (53.16%) patients were between the ages of 36 and 55, followed by 81 (42.63%) patients between the ages of 18 and 35, and 8 (4.21%) patients were over the age of 55. The patients were 38.88 years old on average, with a standard deviation of 8.71 years. The average age was 38 years.

**Table 1:** Age-wise clinical pathology n - Number of cases, % - Percentage

Age categories (Years)	Clinical diagnosis: n (%)			Total	%
	Apical periodontitis	Abscess	Periapical granuloma		
18 – 35	32 (39.5)	42 (51.8)	7 (8.6)	81	42.63
36 – 55	39 (38.6)	53 (52.5)	9 (8.9)	101	53.16
> 55	4 (50.0)	4 (50.0)	0	8	4.21
Total	75 (39.5)	99 (52.1)	16 (8.4)	190	100

In Table 2, the proportion of severe cases was significantly higher in abscesses compared to other diagnosis categories. The difference in the proportion of patients according to the type of resorption in three clinical diagnosis categories was statistically significant with a p-value of 0.00022 using Pearson’s Chi-square test. 

**Table 2:** Distribution of cases according to the type of resorption in each category of clinical diagnosis n - Number, % - Percentage

Clinical diagnosis	Type of resorption: n (%)			Total	P value
	No	Moderate	Severe		
Apical periodontitis	13 (17.33)	47 (62.67)	15 (20)		0.00022
Abscess	12 (12.12)	34 (34.34)	53 (53.54)	99	
Periapical granuloma	3 (18.75)	5 (31.25)	8 (50)	16	
Total	28 (14.74)	86 (45.26)	76 (40)	190	

In Table 3, the proportion of severe cases was significantly higher in abscess and periapical granuloma compared to apical periodontitis. The difference in the proportion of patients according to the type of resorption in three categories of radiological diagnosis was statistically significant with a p-value of 0.0058 using Pearson’s Chi-square test.

**Table 3:** Distribution of cases according to the type of resorption in each category of radiographic diagnosis n - Number, % - Percentage

Radiographic diagnosis	Resorption: n(%)			Total	P value
	No	Moderate	Severe		
Apical periodontitis	8 (15.09)	34 (64.15)	11 (20.75)	53	0.0058
Abscess	19 (16.1)	45 (38.14)	54 (45.76)	118	
Periapical granuloma	1 (5.26)	7 (36.84)	11 (57.89)	19	
Total	28 (14.74)	86 (45.26)	76 (40)	190	

In Table 4, the proportion of severe cases was significantly higher in abscess and periapical granuloma compared to other categories. The difference in the proportion of patients according to the type of resorption in the categories of histology diagnosis was statistically significant with a p-value of 0.00011 using Pearson’s Chi-square test.

**Table 4:** Distribution of cases according to the type of resorption in each histopathological diagnosis category n - Number, % - Percentage

Histological diagnosis	Resorption: n (%)			Total	p-value
	No	Moderate	Severe		
Chronic abscess	2 (4.76)	13 (30.95)	27 (64.29)	42	0.00011
Chronic inflammation	25 (20.49)	65 (53.28)	32 (26.23)	122	
Periapical granuloma	1 (7.14)	5 (35.71)	8 (57.14)	14	
Radicular cyst	0 (0)	3 (25)	9 (75)	12	
Total	28 (14.74)	86 (45.26)	76 (40)	190	

Table 5 shows the correlation between the number of clinical and radiological resorption. The association between the clinical presence of resorption with radiologically moderate or severe type resorption and the absence of the resorption with radiologically mild type of resorption was statistically significant, as indicated by a p-value < 0.00001 using Pearson’s Chi-square test.

**Table 5:** Distribution of cases according to clinical and radiological resorption n - Number, % - Percentage

Clinical Resorption	Radiological resorption: n (%)			Total	P value
	No	Moderate	Severe		
Present	3 (4.23)	24 (33.8)	44 (61.97)	71	< 0.00001
Absent	25 (21.01)	62 (52.1)	32 (26.89)	119	
Total	28 (14.74)	86 (45.26)	76 (40)	190	

## Discussion

Physiological or pathological processes that cause the loss of mineralized tissues like dentin, cementum, and alveolar bone are known as apical root resorption [1]. The predentin layer and the odontoblast layer protect the mineralized tissues found on the inner sides of the root canal, while the pre-cementum, cementoblasts, and periodontal ligament protect the tissues on the outer surfaces. Under normal circumstances, these barriers impede the resorption of these tissues [6]. However, multinuclear cell colonization of denuded tissues can lead to the mineralization of some of these structures, as well as the dislodgement and/or damage of the precementum caused by periapical pathologies, traumatic injuries, pressure/mechanical stimulation, neoplastic conditions, systemic disorders, and idiopathic [7].

The main diagnostic method for root resorption is radiographic examinations that are focused on other clinical conditions. Root resorption is a clinical condition without any symptoms. Additionally, if a diagnosis is made too late, there are very few possibilities of saving the damaged tooth [5]. As apical root resorption is one of the most common phenomena seen in periapical pathologies, diagnosing it earlier along with periapical pathologies will not only help to improve treatment but also help in prognosis.

In order to determine the apical root resorption (ARR) associated with periapical diseases, an attempt was made to examine and appraise the effectiveness and precision of digital intraoral periapical radiography in this study. The study included RVG pictures of 190 patients who met the inclusion criteria and had inflammatory periapical diseases like periapical cysts, periapical granulomas, and periapical abscesses. The presence or absence of resorption in the apical region of the root was assessed in these individuals. Clinical resorption of the extracted teeth was assessed, and RVG pictures for the same were compared.

Among 190 patients with periapical pathologies, in the 18-35 year category, there were a maximum of 42 cases with abscesses, followed by 32 cases with apical periodontitis. While, in the age group 36-55 years, there were 53 cases of abscess, 39 cases of apical periodontitis, and nine cases with periapical granuloma, and in the >55 years category, there were four cases with apical periodontitis and abscess each (Table 1). From the above, it was concluded that the maximum periapical pathologies were in the age group of 36 to 55 years, which was consistent with the study by Gbadebo et al. [11], where the patients analyzed for periapical pathologies were 17 to 57 years with the mean age of 32±11.7 years.

The maximum number of cases in the 18-35 years category showed moderate 35 to severe 34 types of resorption, and 12 cases showed no resorption. Similarly, in the 36-55 years category, only 15 had no resorption and the rest had moderate 47 to severe 39. In >55 years, there were four cases with moderate resorption, while three had severe resorption and one had no resorption, suggesting that there were maximum cases of severe resorption in the age category of 36-55 years followed by cases of moderate resorption in the age category of 18-35 years. This means that, in all groups, most of the patients showed moderate to severe resorption. According to the literature, adults who are older are more susceptible to root resorption in which bone gets more thick, avascular, and aplastic while the periodontal membrane becomes less vascular, aplastic, and narrower. Additionally, the adult population has been found to have a high number of resorbed lacunas and fewer repair zones, which results in root resorption [1]. However, in the present study, since a similar pattern of resorption was seen in all groups, the probable cause could be the periapical inflammatory lesions rather than the physiological causes of aging. Similar findings have been presented by Wei et al. [10], who found that apical root resorption with periapical pathologies was higher in young age followed by middle age followed by old age.

For the diagnosis of periapical lesions, the following signs and symptoms were taken into account: a history of pulpal pain, the presence of pain at the time of examination, sensitivity to percussion, the presence of intraoral or extraoral swelling, sensitivity to hot and cold liquids, severely decayed teeth and root pieces [12]. In the present study, 75 were clinically diagnosed as apical periodontitis, of which 47 showed moderate resorption, followed by 15 with severe resorption and 13 without resorption. There were 99 cases diagnosed with acute and chronic abscess, of which 53 showed severe resorption, 34 showed moderate resorption, and 12 did not. There were 16 cases of periapical granuloma, of which eight showed severe resorption, five had moderate resorption, and three did not. In general, there were 86 cases with moderate resorption, followed by 76 with severe resorption and 28 without resorption. Most of the cases clinically diagnosed were abscesses, and also the overall proportion of severe resorption was significantly higher in abscesses compared to other diagnosis categories. This finding was consistent with the study by Alam et al. [13], in which apical root resorption was also higher in periapical abscesses followed by periapical cyst and granuloma, and apical periodontitis. However, in a study by Wei et al. [10], a statistically significant difference in apical root resorption was found between periapical granuloma and cyst, periapical abscess, and apical periodontitis [10].

The literature suggested that digital intraoral periapical radiography is preferred as a diagnostic tool to assess apical root resorption [9,14]. In the present study also, 71 cases showed the presence of resorption by the clinical method, of which a maximum, that is, 44 showed severe radiological resorption, 24 with moderate resorption, and three without resorption. That is, most 71 cases showed resorption clinically and also showed severe resorption radiographically. However, Schröder et al. [15], found that, compared to the conventional method, the digital radiography imaging method discovered more cavities, regardless of the size of the simulated external root resorption cavities. El-Angbawi et al., in their study, concluded that digital radiographs were the most accurate method to measure apical root shortening [16].

In the present study, the findings of the three observers (one senior radiologist and two post-graduate students) were also compared regarding the evaluation of apical root resorption by checking the morphology of the root surface, width of pdl space, and continuity of the lamina dura on digital radiographs and it was judged to be statistically in fair agreement.

Limitations

The sample in the study is limited to 190 patients. A larger sample size would be desirable so as to substantiate the results. Also, digital radiographs have limitations so histopathological examination can be carried out for the accurate diagnosis of external root resorption, especially when manifested as small defects located on the buccal or lingual surfaces.

## Conclusions

In the present study, patients clinically diagnosed as periapical abscess, periapical granuloma and apical periodontitis, were also diagnosed similarly on radiographic evaluation suggesting that clinical diagnosis of periapical lesions is comparable to the radiographic diagnosis. Most of the cases in the present study had same clinical and radiographical diagnosis but histologically, it showed chronic inflammation on the basis of presence of inflammatory cells only, which is not suggestive of proper a histological diagnosis. Hence, it can be concluded that digital intraoral periapical radiograph’s is accurate in determining and confirming the periapical pathologies, even without doing the histological examination of periapical tissue. Also, maximum patients clinically showing apical root resorption also showed moderate and severe root resorption on radiographic evaluation. Hence, on radiographic examination, digital intraoral periapical radiograph was found to be accurate in determining the periapical apical pathologies and apical root resorption, as most of the cases in the present study had same clinical and radiological diagnosis along with presence apical root resorption clinically and radiographically.
